# Global biogeography of *Bifidobacterium infantis*: stratification and precision probiotic design

**DOI:** 10.1080/19490976.2026.2732532

**Published:** 2026-09-15

**Authors:** Xilong Deng, Jingpeng Yang

**Affiliations:** a State Key Laboratory of Microbial Technology, School of Food Science and Pharmaceutical Engineering, Nanjing Normal University, Nanjing, Jiangsu, China

**Keywords:** *Bifidobacterium longum*, *Bifidobacterium infantis*, infant, genomic atlas, distinct species, geographically matched therapeutics

## Abstract

*Bifidobacterium longum* and *B. infantis* are recognized as pivotal pioneer colonizers in the infant gut, playing instrumental roles in supporting growth, immune maturation, and pathogen resistance. Despite their significance, current genomic understanding is heavily biased toward a limited pool of historical isolates from high-income countries (HICs), leaving the natural diversity of strains circulating in low- and middle-income countries (LMICs) largely uncharted. To address this gap, Shao et al. constructed an unprecedented global genomic atlas comprising 4098 high-quality genomes from 48 countries, thereby expanding LMIC representation by 12- to 17-fold. Through high-resolution phylogenomic and functional analyses, the authors not only delineated *B. infantis* and *B. longum* as distinct species with divergent evolutionary histories but also uncovered profound biogeographic stratification and diet-driven metabolic adaptations at the strain level. These findings challenge the suitability of current probiotic strains and establish a robust scientific blueprint for developing next-generation, geographically matched therapeutics tailored to diverse infant populations.

The assembly of the infant gut microbiome during early life is a critical determinant of immune maturation, metabolic programming, and long-term health trajectories.^1^ ^,^ ^2^ Within this context, *Bifidobacterium* species serve as keystone pioneer colonizers, often constituting over 60% of the total microbial community in infants—dominated by species such as *B. longum*, *B. infantis*, and *B. breve*—before undergoing a significant decline in adulthood (<10%) and further diminishing with aging.^3^ Despite their physiological importance, precise understanding of the *Bifidobacterium* genus has been hindered by two persistent challenges: long-standing taxonomic ambiguity, where *B. infantis* has historically been classified as a subspecies of *B. longum*, and a severe representation bias in genomic resources, which are overwhelmingly derived from industrialized nations while neglecting strains naturally circulating in low- and middle-income countries (LMICs). To address these gaps, Shao et al. analyzed a comprehensive genomic atlas comprising over 4000 high-quality genomes. Through phylogenomic reconstruction and whole-genome average nucleotide identity (ANI) metrics, Shao et al. provide robust evidence supporting the reclassification of *B. infantis* and *B. longum* as distinct species, and resolve the *B. longum* complex into three subspecies: the canonical *B. longum* subsp. *longum*, the animal-associated *B. longum* subsp. *suis*, and a novel subspecies bearing the provisional designation “X” (*B. longum* subsp. “X”; hereafter BX). Because “X” is a placeholder adopted pending formal nomenclatural description and has not been validly published under the International Code of Nomenclature of Prokaryotes (ICNP), it is cited in quotation marks throughout this comment. This lineage had been identified earlier by Arzamasov et al., who designated it *B. longum* “Bl. nov.” based on phylogenomic reconstruction and ANI analysis and provided extensive functional characterization through curated reconstruction of carbohydrate-utilization pathways coupled with experimental validation: a representative strain was uniquely capable, among the tested *B. longum* strains, of growth on soluble starch and pullulan, while HPLC-based glycoprofiling confirmed inefficient utilization of human milk oligosaccharides (HMOs), consistent with the genomic absence of HMO and other host-derived glycan utilization pathways.^4^ The divergence in interim nomenclature between the two studies underscores that a stable, formally validated name for this subspecies remains to be established. Building on this prior work, Shao et al. confirmed the enrichment of this subspecies in infants from industrialized regions using a substantially expanded genomic atlas and further corroborated its specialized metabolic capacity for starch utilization.^5^

Through pan-genomic reconstruction and metabolic pathway analysis across a substantially expanded genomic atlas, Shao et al. corroborated the previously established metabolic divergence between *B. infantis* and *B. longum* at unprecedented scale and population-level resolution. The specialization of *B. infantis* for breast milk-derived substrates, including human milk oligosaccharides (HMOs) and urea, had been documented in earlier experimental and genomic studies, as had the comparatively broader repertoire of *B. longum* for plant-derived glycan catabolism. Notably, with respect to riboflavin (vitamin B2) metabolism, many *B. infantis* genomes encode a complete de novo biosynthesis pathway in addition to uptake mechanisms, whereas *B. longum* generally retains only uptake or salvage capacity^6^; this reflects a difference in vitamin biosynthetic capacity rather than an adaptation to utilize breast-milk-derived riboflavin. The principal novelty of Shao et al.'s analysis thus lies not in the discovery of these metabolic distinctions per se, but in their systematic confirmation across a globally diverse collection of over 4000 genomes, providing population-level resolution of these long-recognized trends.

This study reported biogeographic stratification within *B. infantis* at the strain level, identifying geographically distinct lineages that harbor lineage-associated differences in genes annotated to broad KEGG metabolic categories. Specifically, South Asian lineages showed enrichment of genes annotated to starch metabolism, East African lineages for vitamin B2 and C metabolism, and West African lineages for vitamin B12 and magnesium metabolism. However, it is important to note that these observations are based on enrichment of individual KEGG KOs and do not, by themselves, establish the presence of complete metabolic pathways or corresponding phenotypes. For instance, the gene annotated as supporting “ascorbate metabolism” in the East Africa 2 lineage (K03077, encoding L-ribulose-5-phosphate 4-epimerase) can also function in L-arabinose, L-xylulose, or related pentose metabolism and therefore does not, by itself, demonstrate vitamin C utilization. Similarly, the enrichment of a gene annotated as amylosucrase (K05341) in South Asian strains does not establish the ability to degrade and utilize starch, which requires distinct extracellular CAZymes; moreover, growth experiments with diverse Bangladeshi and Malawian *B. infantis* isolates did not support starch utilization.^4^

The gene annotated as evidence for vitamin B12 metabolism (K01552) encodes a shared ATP-binding component of an energy-coupling factor transporter whose substrate specificity requires identification of the associated transporter components and genomic context; furthermore, no vitamin B12-dependent enzymes have been described in *B. infantis* to date, making the biological relevance of B12 salvage unclear. These results should therefore be interpreted as lineage-associated differences in genes annotated to broad metabolic categories whose functional significance remains to be established through pathway-level reconstruction and experimental validation. The hypothesis that these associations reflect dietary co-evolution is intriguing but premature in the absence of such validation. Notably, earlier work by Barratt et al. provided experimentally investigated evidence of geography-associated functional variation in *B. infantis*, including the β-glucoside- and N-glycan-utilization loci (*bgl* and *ngl* gene clusters) characterized in the Bangladeshi strain Bg_2D9, which demonstrated superior fitness in gnotobiotic mouse models.^7^ Together, these experimental observations and the expanded genomic survey by Shao et al. establish geography-associated, strain-level metabolic variation within *B. infantis* as a genuine biological phenomenon; whether such variation can be translated into a rational strain-selection strategy, however, is a question that must first be framed by the clinical context.

Striking epidemiological disparities exist in *B. infantis* prevalence, which dominates the infant gut microbiome in low- and middle-income countries (LMICs) at rates approaching 80%, yet is scarcely detected (<2%) in infants from industrialized nations.^8^ This disparity is primarily attributed to the cumulative impact of industrialized lifestyle factors—such as antibiotic exposure, formula feeding, cesarean delivery, and early weaning—which collectively erode the ecological niche essential for *B. infantis* colonization. It is against this clinical backdrop—rather than on metabolic evidence alone—that the rationale for geographically matched probiotics should be evaluated. Read together, the two threads form a coherent narrative: the prevalence disparity between LMICs and industrialized regions defines the clinical problem, explaining why geographically informed probiotic design is needed, whereas the geography-associated metabolic variation provides the scientific basis for why such matching may be achievable. These complementary lines of evidence support geographically matched probiotic candidates as a hypothesis warranting further pathway-level and experimental investigation^9^ (Figure 1). Concurrently, the disappearance of this pioneer species is hypothesized to contribute to the rising incidence of “diseases of civilization,” including autoimmune and metabolic disorders, in industrialized settings.^10^ Furthermore, this study raises questions about the suitability of widely used commercial probiotics. However, the characterization of strains such as EVC001 as possessing “incomplete HMO-utilization gene clusters” is factually inaccurate. Comparative genomic analyses place EVC001, together with the type strain ATCC 15697, among the *B. infantis* strains with the most extensive HMO-utilization machinery.^11^ EVC001 carries a complete H1 cluster encoding multiple HMO transporters and glycoside hydrolases, a complete *lnp* cluster including the GltABC transporter involved in LNT uptake—which is absent from many natural isolates^4^—and two FL clusters encoding transport systems for fucosylated HMOs.^12^ Moreover, variation or partial loss of individual HMO-associated genes in *B. infantis* does not necessarily translate into diminished HMO-utilization capabilities, owing to the substantial functional redundancy among the transporters involved in HMO metabolism. It should also be noted that the HMO utilization gene analysis in Shao et al. contains errors: the study concluded that LNB/GNB utilization is not conserved in *B. infantis* compared with *B. longum* and *B. suis*, but this conclusion appears to result from omission of the alternative LNB/GNB transporter encoded by Blon_0883–0885,^13^ causing strains lacking GltABC to be misclassified as non-utilizers despite retaining an alternative LNB/GNB uptake system. That said, the broader argument that commercial probiotic strains—largely derived from historical mid-20th century isolates—exhibit limited overlap with the genetic diversity of contemporary natural microbiota, and that population-specific probiotic selection may be beneficial, is reasonable. However, given that Shao et al.'s analysis is predominantly gene-level, based on KEGG functional assignments with the noted limitations in HMO pathway reconstruction, it does not provide sufficient functional resolution to identify “precise genomic and metabolic compatibility” or to nominate specific geographically matched strains. These respective claims should therefore be understood as approximate and preliminary conjectures requiring further pathway-level reconstruction and experimental validation.

**Figure 1.**
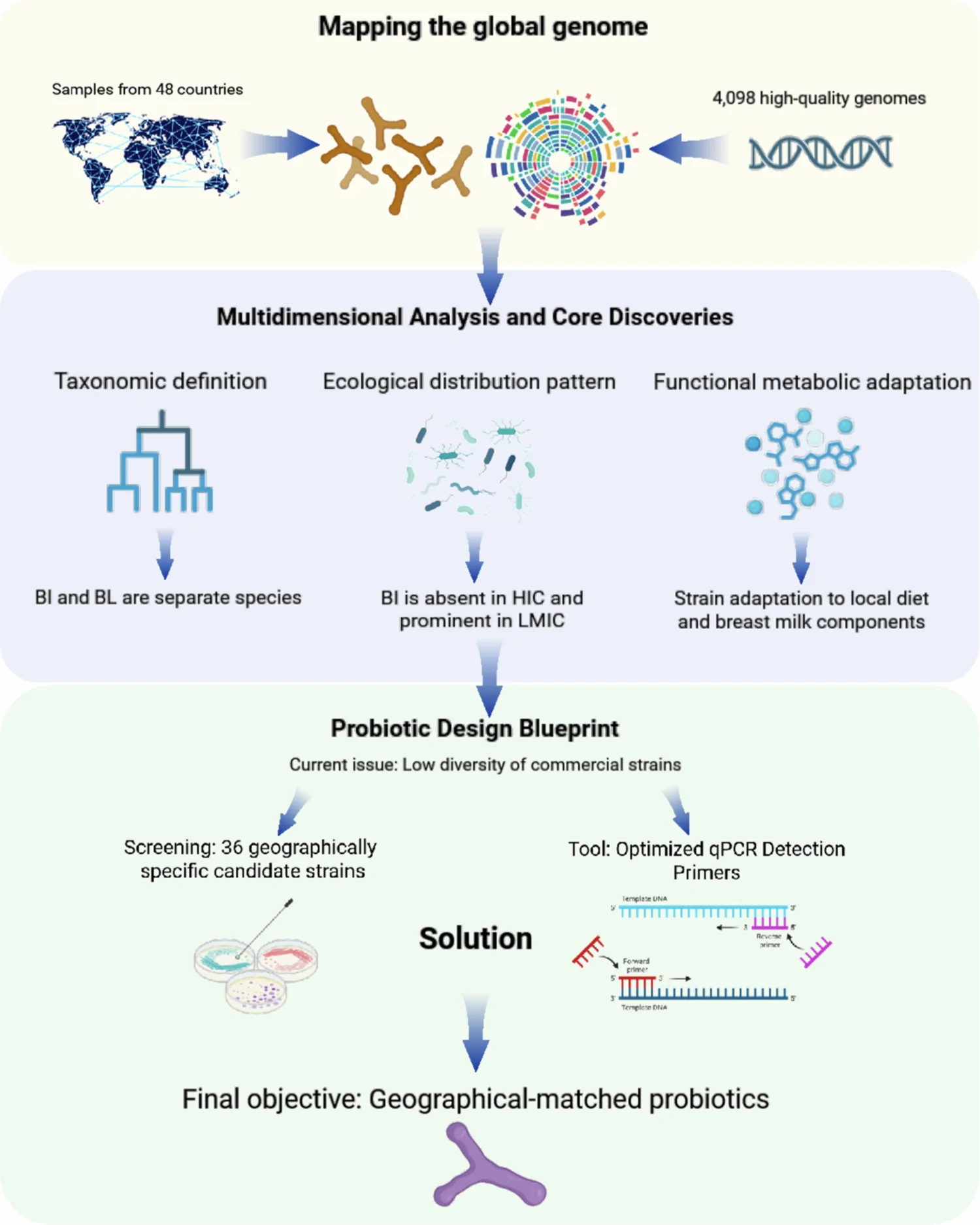
Global genomic atlas of *Bifidobacterium longum* (BL) and *B. infantis* (BI): biogeographic stratification and a blueprint for precision probiotic design.

Beyond its fundamental scientific insights, this study offers potentially valuable methodological and translational resources. Leveraging newly identified conserved genomic loci—most notably the urease cluster—the authors designed a suite of qPCR assays intended to differentiate among *B. infantis*, *B. longum*, and the novel subspecies BX. In their in silico analysis, these candidate primer targets displayed improved specificity and reduced cross-reactivity relative to conventional primers targeting variable human milk oligosaccharide (HMO) utilization genes. However, the new assays were evaluated primarily in silico and should therefore be regarded as candidate assays requiring experimental validation across diverse isolates, closely related *B. longum* subspecies, and complex microbiome samples; the claim that they “markedly outperform” existing primers has thus not been fully established. This caveat is particularly important because urease genes are not unique to *B. infantis* and have also been reported in closely related lineages, including *B. longum* subsp. *iuvenis* and subsp. *suis*.^14^ An important limitation of the underlying genomic dataset also warrants mention: a large proportion of the analyzed genomes were short-read metagenome-assembled genomes (MAGs) retained at ≥90% estimated completeness. Although this threshold may be sufficient for broad phylogenomic analyses, it is less reliable for assessing the presence or absence of individual genes and large multigene loci. HMO-utilization loci such as the H1 cluster are frequently fragmented or incompletely recovered even in short-read assemblies of isolate genomes, and this problem is likely more pronounced in MAGs. Consequently, the apparent absence of nanH2—which encodes an α-sialidase^15^ and has previously served as a PCR marker for *B. infantis* ^16^—as well as the broader conclusion that HMO utilization capacity is not conserved in *B. infantis*, may partly reflect assembly incompleteness rather than genuine biological variation. Notably, nanH2 was detected in 94.1% of *B. infantis* genomes, and all genomes lacking this gene were MAGs rather than isolate genomes—a pattern consistent with this technical explanation. With these caveats acknowledged, the publicly accessible genomic repository established by the authors—comprising 115 cultivated isolates, 2875 MAGs, and a curated panel of 36 geographically specific candidate probiotic strains—remains a valuable resource that may accelerate microbiome research and inform preliminary exploration of population-matched therapeutic candidates.

Despite its comprehensive scope, this study is subject to several notable limitations. First, the primary conclusions are largely derived from computational inferences, lacking systematic phenotypic validation through metabolomic profiling, *in vivo* models, or clinical intervention studies. Second, despite the expanded genomic representation from low- and middle-income countries (LMICs), significant geographic gaps persist; regions such as Latin America, Oceania, and specific Indigenous populations remain under-sampled. Furthermore, the lack of integration with host genetic data (e.g., secretor status, milk composition) and harmonized socio-environmental metadata constrains the attribution of causality, hindering a definitive dissection of the mechanisms driving biogeographic stratification. To address these gaps, future investigations should prioritize three key trajectories: first, employing integrated culturomics and metabolomics to empirically validate the metabolic functions of geo-specific lineages; second, leveraging longitudinal cohorts to elucidate the relative contributions of vertical transmission versus environmental selective pressures through host-microbe co-evolutionary analyses; and third, conducting multicenter, randomized controlled trials (RCTs) to rigorously benchmark the colonization fitness, safety profiles, and clinical efficacy of “geographically matched probiotics” against conventional commercial strains.

Shao et al. provide a valuable large-scale description of the geographic population structure and gene-content variation within infant-gut *Bifidobacterium*, offering a useful foundation upon which future microbiome interventions may build. However, these observations do not, by themselves, establish a paradigm shift toward precision-matched probiotics. The biogeographic stratification of *B. infantis* documented in this study is suggestive but does not per se demonstrate host–microbe co-evolution, as the observed patterns may alternatively reflect bacterial population structure, founder effects, selective sweeps, or sampling biases; demonstrating co-evolution would require more elaborate evolutionary analyses linking microbial genomic variation with host genomic data. Moreover, the study does not show that population-derived strains are necessarily more effective than widely used probiotic strains, nor does it provide sufficient functional or clinical evidence to substantiate the concept of “precision biotherapeutics.” Previous work, including the improved fitness of the Bangladeshi strain Bg_2D9 under specific experimental conditions,^7^ supports the possibility that population-associated strain differences can affect probiotic performance; however, such studies remain limited, and the broader field of population-specific probiotic design is still at an early stage. On balance, the available evidence supports incorporating geographic and strain-level genomic diversity when prioritizing strains for functional evaluation, and the notion that next-generation probiotics may benefit from being adapted to the populations they are intended to serve should be presented as a promising hypothesis warranting substantial future work—including pathway-level functional characterization, experimental validation, and controlled clinical trials—rather than an established directive.

## Data Availability

Not applicable.
